# Transarterial Microembolization in Neuropathic Postmastectomy Pain

**DOI:** 10.1007/s00270-026-04354-1

**Published:** 2026-02-09

**Authors:** F. Frenzel, A. Taheri Amin, P. Minko

**Affiliations:** 1 Clinic of Diagnostic and Interventional Radiology, Saarland University Hospital, Homburg, Germany; 2 Department of Diagnostic and Interventional Radiology, Charité-Universitätsmedizin Berlin, Freie Universität Berlin and Humboldt-Universität Zu Berlin,Campus Virchow-Klinikum (CVK), Augustenburger Platz 1, 13353 Berlin, Germany; 3 Department of Diagnostic and Interventional Radiology, University Hospital Duesseldorf, Medical Faculty, D-40225 Duesseldorf, Germany

**Letter to the Editor**

Neuropathic chest wall pain is a known complication after breast surgery, affecting 20–50% of patients, with odds increased 3.1-fold after axillary lymph node dissection [1]. Regional techniques such as nerve blocks may provide temporary benefit, yet their durability is limited, with no improvement in health-related quality of life [2]. Although neuropathic pain differs clinically from pain in osteoarthritis, experimental studies have demonstrated that nerve injury–related pain is similarly associated with increased neovascularization [3].

Given the overlapping pathophysiological features, we explored the use of transarterial microembolization in patients with neuropathic breast and chest wall pain.

**Fig. 1:**
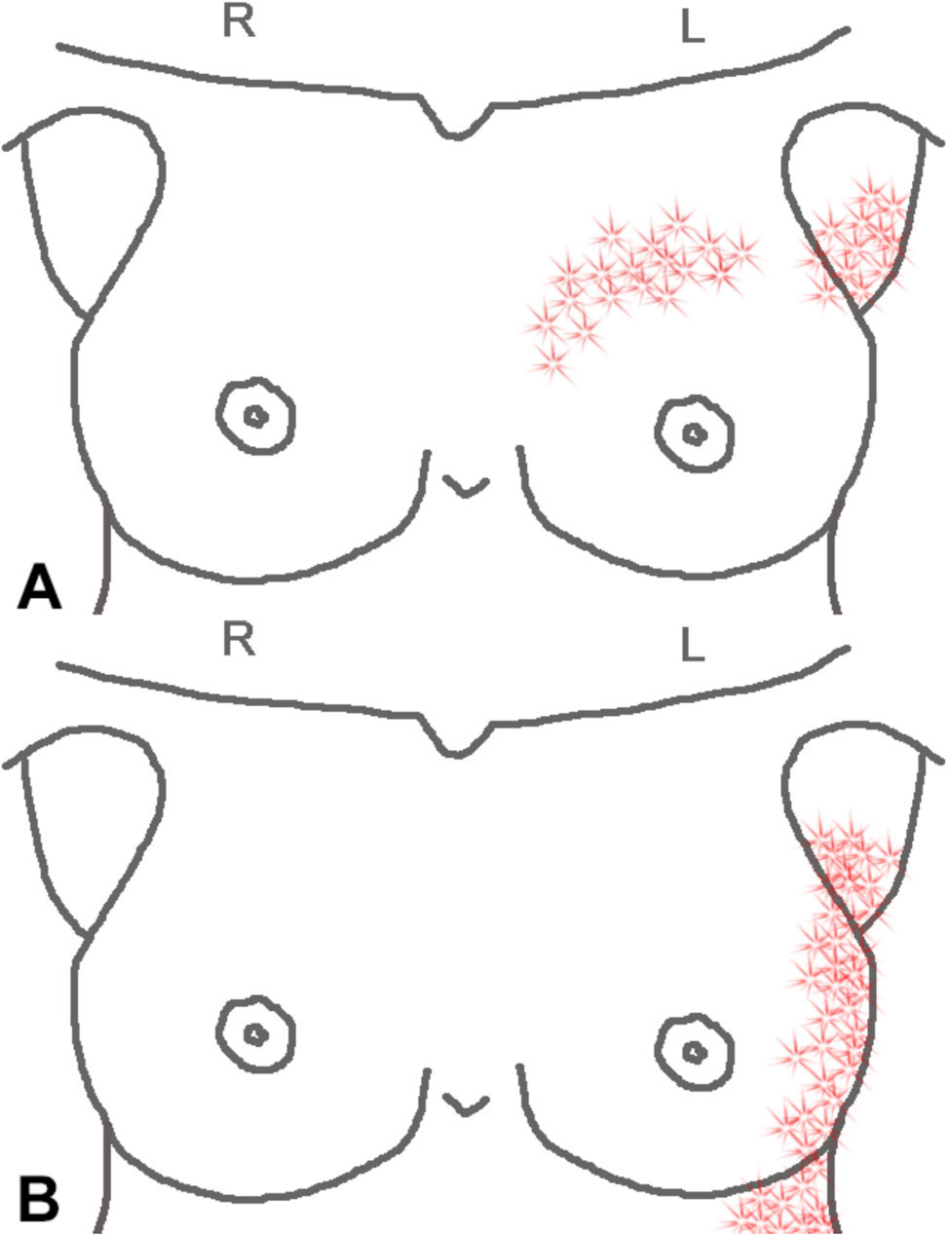
Schematic drawings of pain locations in the treated patients. **A** 53-year-old female suffering from neuropathic breast pain and pain in the axilla. The patient had received surgery 9 years ago followed by radiotherapy. Right beneath the scar in the left axilla, a known nodular mass was palpable, resulting in severe pain of the patient. **B** 40-year-old female suffering from neuropathic pain in the lateral half of the breast, axilla and lateral thorax after trauma in the youth

Patient A is a 53-year-old woman with neuropathic pain in the left breast and axilla, accompanied by sensory irritation, after breast-conserving surgery with axillary dissection and radiotherapy. Despite more than six months of Hyoscine butylbromid 10 mg four times daily and Metamizol 500 mg up to eight times daily, no improvement occurred. Baseline Numeric Rating Scale (NRS) scores was 10 in affected areas (Fig. 1A).

**Fig. 2:**
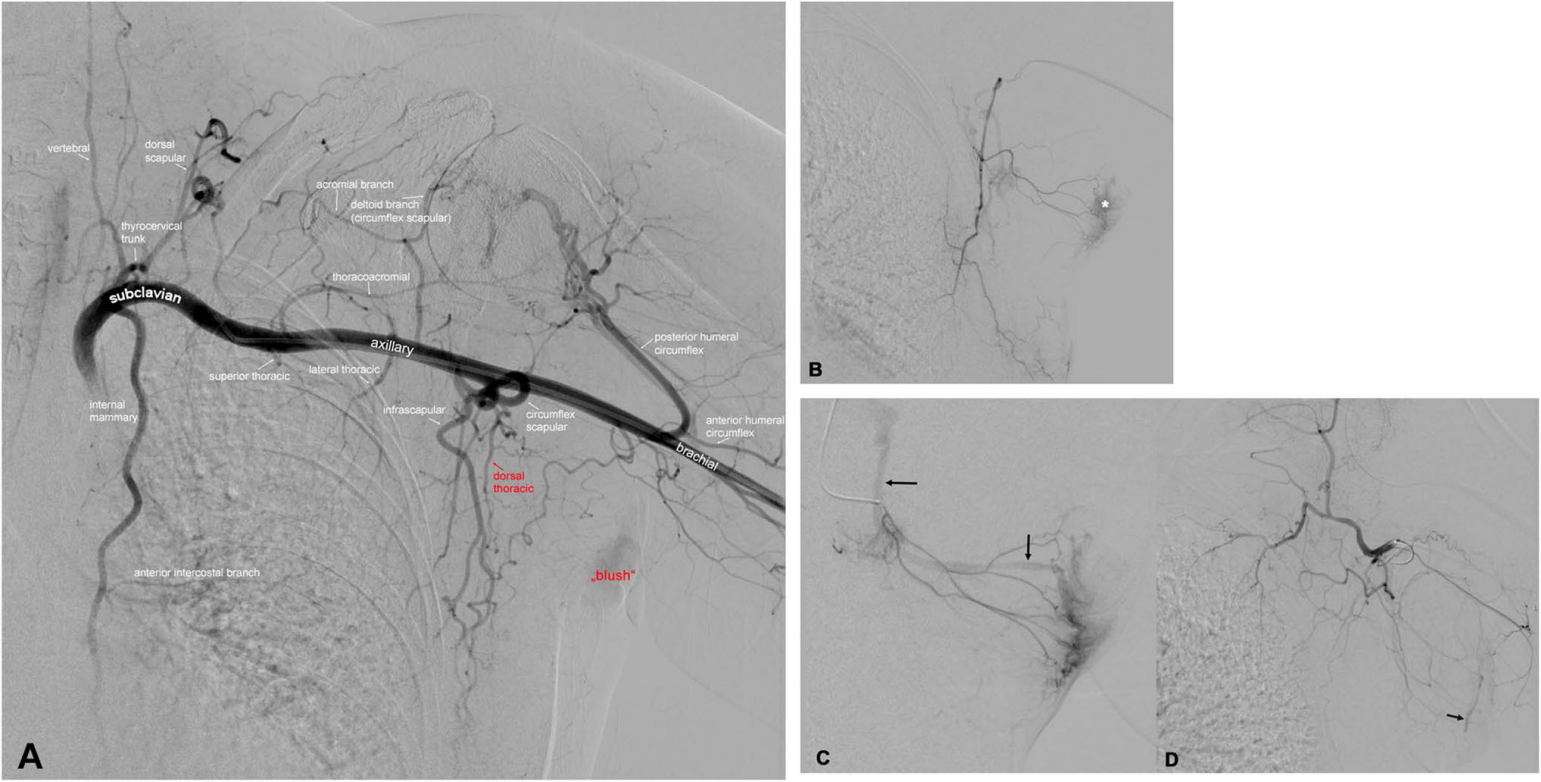
Digital subtraction angiography (DSA) of the axillary artery and side branches before and during embolization in a 53-year-old-female. **A** DSA of the axillary artery showing an overview of the axillary artery’s anatomy with side branches as described. **B** Selective DSA after cannulation of the dorsal thoracic artery before embolization. A hypervascularization (“blush”), which correlated well to the patient’s known pain sensation is visible and marked with an asterisk. **C** Superselective DSA after further advancement of the microcatheter inside a branch of the dorsal thoracic artery supplying the hypervascularization in the axilla. Early venous flow (black arrows) is a hallmark of the chronic local inflammation. The known pain sensation was provoked after injection of contrast medium. **D** DSA after embolization with 2.5 mL of Imipenem/Cilastatin diluted in 7 mL of contrast agent showing diminished hypervascularization and preservation of an efferent vein (arrow). At this time point the patient did not feel the initially provoked pain in the axilla anymore

The procedure was performed via a retrograde transradial approach under local anesthesia and ultrasound guidance. A 5F sheath (Check-Flo, Cook Medical, USA) was placed, followed by systemic heparinization (1,250 IU). Diagnostic angiography of the axillary artery was conducted using a 4F catheter (Glidecath, Terumo, USA) followed by manual injection of iodinated contrast medium (Imeron 300, Bracco, Italy). Embolic material was prepared by diluting 500 mg of Imipenem/Cilastatin (Prepenem, Merck & Co., USA) in 7 mL of the contrast medium mentioned above. The ostium of the subscapular artery was probed using a 4F Judkins catheter (JR 3.5, Cordis, USA). Then superselective catheterization of the dorsal thoracic artery was achieved using a 1.7F microcatheter (Pursue, Merit Medical, USA) and a 0.014-inch guidewire (Radifocus, Terumo, USA (Fig. 2A). Superselective DSA revealed focal hypervascularization accompanied by the patient’s characteristic pain. Embolization was performed using 2.5 mL of the suspension mentioned above. An ice pack was applied over the treated area to reduce skin perfusion. Post-embolization DSA confirmed pruning of the target vessels with preserved flow in the parent vessels (Fig. 2B-D). The radial puncture site was secured with a compression device (TR Band, Terumo, USA).

The patient experienced immediate and complete pain relief, with NRS decreasing from 10 to 0 in both regions. She was also able to touch the upper breast and axilla without sensory irritation for the first time in years. The complete resolution of pain has persisted to date, four years after the intervention. All pain medication was discontinued after the procedure.

Patient B is a 40-year-old woman with neuropathic pain in the left lateral chest wall following shoulder trauma in adolescence. Symptoms had persisted despite more than six months of conservative therapy, including Methocarbamol 750 mg twice daily and Pregabalin 150 mg three times daily. Baseline NRS was 8 in the affected regions (Fig. 1B).

**Fig. 3:**
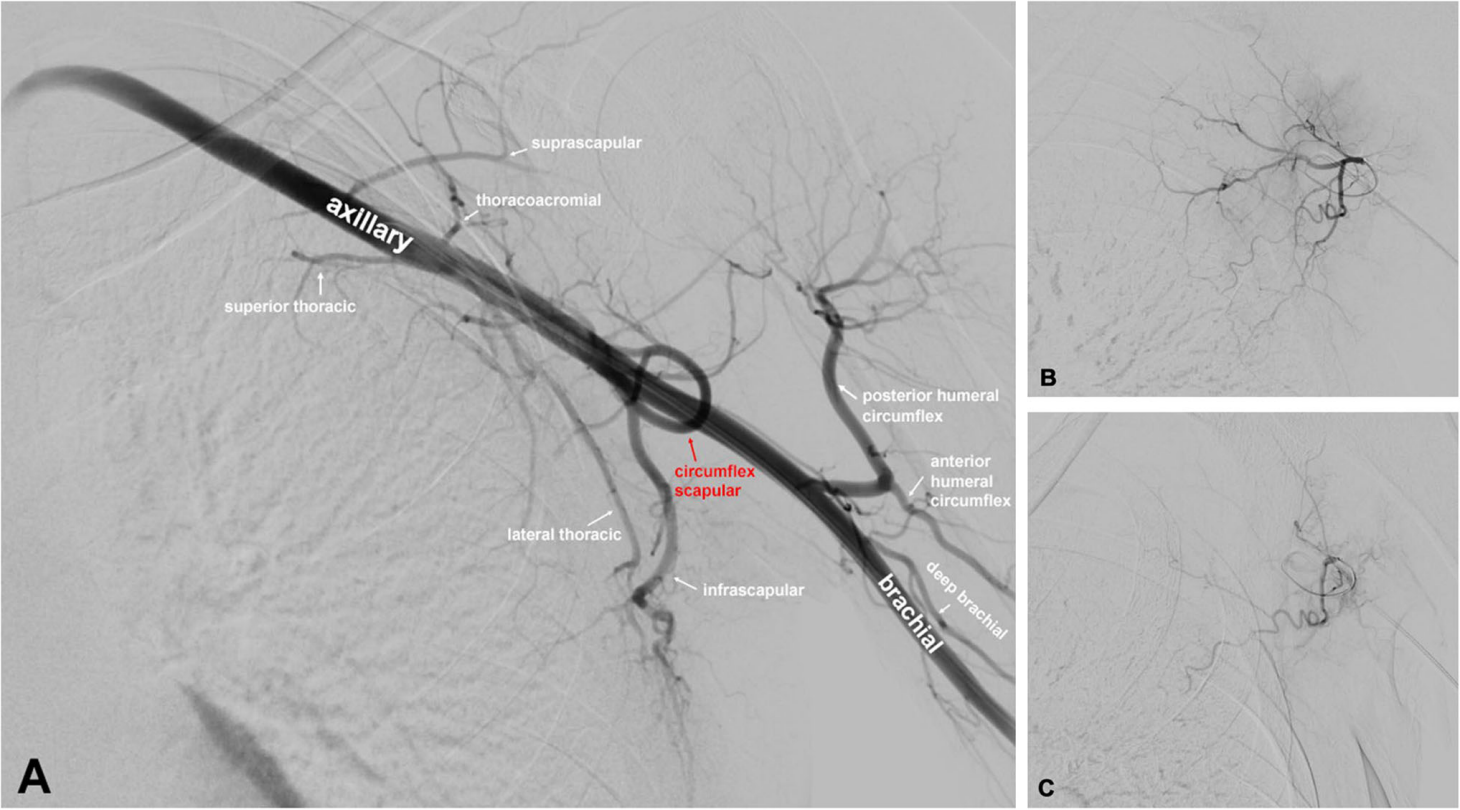
Digital subtraction angiography (DSA) of the axillary artery and side branches before and during embolization in a 40-year-old-female. **A** DSA after injection of contrast medium showing an anatomical overview of the axillary artery and its side branches as described. **B** Selective DSA of the circumflex scapular artery after selective catheterization before embolization. DSA shows a slight hypervascularization in the axilla and lateral thorax wall. The injection of contrast medium provoked pain, which correlated with the known pain sensation usually felt by the patient. **C** DSA after embolization with 2.5 mL of Imipenem/Cilastatin diluted in 7 mL of contrast agent demonstrating a ceased hypervascularization of the target vessels with patent branches of the parenting circumflex scapular artery. The initially provoked pain in the axilla upon contrast medium administration was not felt anymore after embolization

All procedural steps were identical to those in Patient A, except that the circumflex scapular artery was probed using a 5F AC1 catheter (Cook Medical, USA) (Fig. 3A). Embolization was again performed with 2.5 mL of the suspension mentioned above (Fig. 3B/C).

The patient reported a decrease in NRS from 8 to 1 immediately after the intervention. Pain gradually increased to an NRS of 3 after one year but remained stable through four years of follow-up. All pain medication was discontinued after the procedure.

Both patients were discharged the following day. Mild skin erythema over the axilla and lateral chest wall developed shortly after the intervention in both patients and resolved within 4–6 h. No other complications were observed.

We observed sustained pain relief following TAME in two patients with neuropathic chest wall pain, with clinical benefit persisting for up to four years. Neuropathic pain typically results from injury to peripheral nerves. Underlying pathophysiological mechanisms involve immune–neural interactions, leading to peripheral and central sensitization [4]. Previous studies have demonstrated that these processes are associated with vascular endothelial growth factor signaling and neovascularization [3]. Because similar mechanisms contribute to osteoarthritis pain, it appeared reasonable to extend the concept of TAME to neuropathic pain.

The arterial supply of the chest wall is extensive and redundant, with overlapping vacular territories. This poses major challenges for TAME: Multiple arteries may contribute to the same pain region and dense anastomoses increase the risk of non-target embolization. Given the exploratory design, the initial strategy was to keep the intervention as simple as possible by embolizing only a single vessel.

In Patient A, whose pain occurred after breast cancer surgery, the dorsal thoracic artery was selected, as it supplies the locoregional lymph nodes and lies along the operative access route [5]. In Patient B, the circumflex scapular artery was targeted, as it represents the primary arterial supply to the shoulder joint, which had previously been injured.

To minimize the risk of non-target embolization, Imipenem/Cilastatin, a temporary embolic agent with established efficacy in TAME, was used. Furthermore, the microcatheter was positioned superselectively to minimize reflux into adjacent vessels and ice packs were placed around the treated area to promote cutaneous vasoconstriction.

This report is limited by the small number of patients and the absence of a control group, precluding definitive conclusions regarding efficacy. However, TAME may represent a promising minimally invasive treatment option for patients with refractory neuropathic chest wall pain. Larger prospective studies are required to confirm these preliminary findings and to establish its safety and clinical efficacy.
